# Prevalence and risk factors for anxiety in patients with early- and middle-stage lung cancer: a cross-sectional study

**DOI:** 10.3389/fpsyg.2024.1413591

**Published:** 2024-08-23

**Authors:** Ruoqi Zhang, Peitong Zhang, Yuejie Lin, Xiuwei Guo, Jing Wang

**Affiliations:** ^1^Department of Oncology, Guang’anmen Hospital, China Academy of Chinese Medical Sciences, Beijing, China; ^2^Graduate College, Beijing University of Chinese Medicine, Beijing, China

**Keywords:** early- and middle-stage, lung cancer, anxiety, incidence rate, risk factors

## Abstract

**Objective:**

Lung cancer is a leading cause of cancer-related morbidity and mortality worldwide, with patients frequently experiencing significant psychological distress, particularly anxiety. Despite the high prevalence of anxiety in patients with cancer, there is limited comprehensive research focusing on the specific factors influencing anxiety in patients with early- and middle-stage lung cancer within the context of Chinese medicine hospitals. Therefore, we aimed to investigate the epidemiology and factors influencing anxiety disorders in patients with early- and middle-stage primary bronchial lung cancer through a cross-sectional study.

**Methods:**

A total of 340 patients with early and middle-stage lung cancer admitted to the outpatient ward of the oncology department at Guang’anmen Hospital from June 2023 to December 2023 were included in this study. Survey data, including the patients’ general condition questionnaire, Generalized Anxiety Scale (GAD-7), Hospital Anxiety and Depression Scale (HADS), and Mental Toughness Scale (CD-RISC-10), were collected and recorded in a database using a two-person input format. Data analysis was performed using SPSS 27.0 software.

**Results:**

Out of the 340 patients with early- and middle-stage lung cancer included in this study, 133 had anxiety, resulting in an overall anxiety detection rate of 39.12%. The chi-square test showed that statistically significant differences in religion, marital status, surgical treatment, tobacco use, and alcohol history between the anxious and non-anxious groups (*p* < 0.05). Moreover, statistically significant differences were observed in *per capita* annual family income, pathological type, VAS score, targeted therapy, treatment stage, and mental toughness level (*p* < 0.001). Other factors were not significantly correlated with anxiety onset. Multivariate logistic regression analysis showed that higher *per capita* family income and completed treatment independently acted as protective factors against anxiety onset in patients with early- and middle-stage lung cancer. Conversely, rare pathological types, increased pain severity, and lower levels of mental toughness were identified as independent risk factors for anxiety onset in these patients.

**Conclusion:**

Anxiety was prevalent in patients with early- and middle-stage lung cancers. Rare pathological types, increased pain severity, and lower levels of mental toughness were independent risk factors for anxiety. Therefore, clinicians and psychologists should pay more attention to patients with rare types of tumors, actively manage their pain symptoms, and consider implementing mental resilience training to improve patients’ mental toughness.

## 1 Introduction

Cancer is one of the leading causes of death worldwide and places a huge burden on patients and their families. According to the IARC Global Cancer Statistics Report (Sung et al., 2021), lung cancer has the highest mortality rate (18.0%) among all cancer types, followed by breast cancer (11.4%). Patients with primary bronchogenic carcinoma often experience different degrees of anxiety disorders because of disease symptoms, treatment toxicity and side effects, fear of disease progression, and heavy economic burdens. Anxiety manifests both physiologically (e.g., tension headaches, poor sleep, and fatigue) and psychologically (e.g., fear, a desire to escape, and a sense of being out of control). Previous studies have investigated the prevalence of anxiety in patients with lung cancer; however, the results remain controversial. Yan et al. (2019) found an anxiety prevalence of 43.5% among 315 patients with lung cancer. Wang et al. (2022) reported that 34.2% of inpatients with lung cancer experienced anxiety. Another study found that the rate of anxiety was as high as 61% among patients with lung cancer on the first day after chemotherapy (Topcu et al., 2023). These discrepancies may be related to different sample sizes, study areas, and psychological assessment scales. Despite these variations, the detection rates of anxiety in patients with lung cancer are higher than those in the general cancer population (10%) and the general population (7%) (Pitman et al., 2018).

Studies have shown that patients with lung cancer with anxiety have a lower quality of life and poorer prognosis than those without anxiety (Arrieta et al., 2013; Vodermaier et al., 2017). However, most previous studies have focused on advanced-stage patients (Blevins et al., 2024; Gonzalez-Ling et al., 2023; He et al., 2022), and few studies have investigated patients with early- and middle-stage lung cancer. Patients with early- and middle-stage lung cancer are more likely to receive radical or palliative surgical treatment, which can result in longer survival or even recovery after effective treatment. Previous studies have surveyed risk factors, such as sex, age, and family income, but the results have been inconclusive. Some studies have found that female patients are more prone to anxiety (Morrison et al., 2017; Obispo-Portero et al., 2022), while others have suggested that anxiety is not related to gender due to the evolving roles of men and women in the society (Yan et al., 2019). The main research view is that younger patients are more anxious than older patients (Chambers et al., 2015), whereas a few studies have reported that older patients are more prone to anxiety (Hong and Tian, 2014). This could be due to the fact that some older patients lack life support and have multiple chronic diseases. Most previous studies on mental disorders have been conducted in Western medical hospitals, with only a few conducted in hospitals practicing Chinese medicine (Yan et al., 2019; Wang et al., 2022). Therefore, it is crucial to explore the risk factors for anxiety in patients with early- and middle-stage lung cancer in hospitals practicing Chinese medicine. This research is significant for early identification and intervention and fills gaps in the relevant research areas.

## 2 Methods

### 2.1 Study design and participants

We conducted a cross-sectional study, recruiting patients with early- and middle-stage primary bronchogenic carcinoma who were admitted to the Oncology Department of Guang’anmen Hospital from June to December 2023. Clinicians and researchers were responsible for recruiting patients when they arrived at Guang’anmen Hospital. Patients who consent to participate in the study completed the questionnaire during their leisure time in the outpatient clinic or oncology ward. It took no more than 30 min to complete the questionnaires. Convenience sampling was used to select patients. The inclusion criteria included the following: (1) diagnosis of primary bronchogenic carcinoma according to pathology and/or cytology; (2) clinical stage I–IIIa; (3) ages between 18 and 80 years; (4) Karnofsky score ≥ 60 and (5) informed consent. The exclusion criteria were as follows: (1) the presence of other malignant tumors, (2) poor comprehension, and (3) impaired vision or hearing. The purpose of the study was explained in detail to patients who agreed to participate.

### 2.2 Procedures

Data were collected using a questionnaire with informed consent from the patients and their family members. In some cases, patients involved their close relatives in the informed consent process, even though they were capable of providing informed consent themselves. The purpose of the study and relevant points of attention were explained by trained investigators before the participants completed the questionnaire. If patients had doubts during the filling-in process, the investigator clarified the questionnaire items face-to-face using consistent language. The questionnaire mainly included demographic information, clinical data, mental toughness levels, and the mental condition of the patients. The investigators individually checked the completeness and accuracy of the completed questionnaires. The results were entered into Epidata 3.0 software by two independent researchers, and the verified information was analyzed using SPSS 27.0 software. Three hundred and forty-two patients were invited to participate, two of whom were excluded because of missing vital information. Ultimately, 340 questionnaires were included in the analysis. All participants provided written informed consent. This study was approved by the Ethics Committee of Guang’anmen Hospital (No.2023-142-KY).

### 2.3 Measures

#### 2.3.1 General survey

The scale was designed by the researchers and included sex, age, household registration, education level, marital status, family income, religion, tumor location, pathological type, clinical stage, degree of differentiation, duration of illness, surgery, treatment phase, KPS score, VAS score, smoking and drinking status, comorbidity with chronic illness, and family history.

#### 2.3.2 Generalized anxiety disorder scale-7

The GAD-7 was designed by Spitzer et al. (2006), which consists of seven symptom items and one symptom-related difficulty item, using a four-point scale (0 = never, 1 = occasional days, 2 = frequent, more than a week in the past 2 weeks, 3 = almost every day). Participants were assessed for their level of anxiety in the last 2 weeks. The total score of the scale was 21 points, with scores interpreted as follows: 0–4 points indicate no anxiety state, 5–9 points indicate mild anxiety, 10–14 points indicate moderate anxiety, and 15–21 points indicate severe anxiety. The GAD-7 scale is widely used in primary care settings to screen for the presence and severity of anxiety symptoms, with a Cronbach’s α coefficient of 0.843 (Gong et al., 2021).

#### 2.3.3 Hospital anxiety and depression scale

The HADS was designed by Zigmond and Snaith (1983) and has the advantages of ease of operation and rapid screening capability. The HADS consists of two subscales, HADS-A (anxiety) and HADS-D (depression), each containing seven items. The scoring system ranges from 0 to 3 for each item. Scores for anxiety or depression were classified as follows: 0–7 indicate a negative mental disorder, 8–10 indicate a mild mental disorder, 11–14 indicate a moderate mental disorder, and 15–21 indicate a severe mental disorder. Higher scores indicate greater levels of anxiety and depression. This scale is an effective tool for detecting anxiety and depression in individuals with physical symptoms.

The prevalence of anxiety was determined using a combination of the GAD-7 and HADS-A scores. Participants were considered to have an anxiety disorder if they scored ≥5 on the GAD-7 scale and ≥8 on the HADS-A scale and were included in the anxiety group. Otherwise, they were included in the non-anxiety group for statistical analysis. The GAD-7 scale was mainly used to evaluate the degree of anxiety: 5–9 for mild anxiety, 10–14 for moderate anxiety, and 15–21 for severe anxiety.

#### 2.3.4 Connor-Davidson resilience scale-10

The CD-RISC was originally proposed by Connor and Davidson (2003) and Campbell-Sills and Stein (2007) reduced it to a 10-item scale. The scale consists of 10 items graded on a five-point scale of 0–4 (0 = not at all, 1 = rarely, 2 = sometimes, 3 = often, 4 = almost always). Resilience is divided into four grades: ≥34, 28–33, 21–27, and ≤20, with the highest possible total score being 40. Higher scores indicate better resilience. The CD-RISC-10 has good reliability and validity and is widely used in the community, outpatient clinics, and other places to assess the level of mental toughness, with a Cronbach’s α coefficient of 0.93 (Alahdab et al., 2020).

### 2.4 Statistical analysis

The questionnaire information was entered into the database by two independent investigators using Epidata 3.0 software. Data were analyzed using SPSS 27.0. Continuous data are expressed as means and standard deviations, while categorical data are expressed as frequencies and constituent ratios. In the univariate analysis, categorical data were tested using the chi-square test, and Fisher’s exact test was used when the chi-square test conditions were not met. Variables with *p* < 0.05 in the univariate analysis were included in the multivariate logistic regression analysis to examine factors related to anxiety in patients with early- and middle-stage lung cancer. All data were tested using a two-sided statistical method, with a significance level set at α = 0.05.

## 3 Results

### 3.1 Basic characteristics of participants

After excluding two patients with missing information, 340 patients with early- and middle-stage lung cancer meeting the inclusion criteria were included in this study. This sample comprised 154 males and 186 females, with a male-to-female ratio of 5:6. The patients ranged in age from 34 to 80 years: 52 were aged 34–49 years, 134 were aged 50–59 years, 100 were aged 60–69 years, and 54 were aged 70–80 years. The clinical stages of all the participants ranged from IA to IIIA, including 212 patients with stage IA, 23 patients with stage IB, 15 patients with stage IIA, 33 patients with stage IIB, and 57 patients with stage IIIA. Pathological types included adenocarcinoma (248 cases), squamous cell carcinoma (29 cases), small-cell carcinoma (52 cases), and rare pathological types (11 patients) (Table 1).

**Table 1:** Demographic characteristics and univariate analysis of participation.

Item	Without anxiety	Mild anxiety	Moderate anxiety	Severe anxiety	Total	*X* ^2^	*p* value
**Gender**							
Male	88 (25.88%)	9 (2.65%)	41 (12.05%)	16 (4.71%)	154 (45.29%)	1.653	0.220
Female	119 (35.00%)	9 (2.65%)	37 (10.88%)	21 (6.18%)	186 (54.71%)		
**Age (years)**							
34~	28 (8.24%)	4 (1.18%)	12 (3.53%)	8 (2.35%)	52 (15.30%)	4.331	0.228
50~	79 (23.23%)	5 (1.47%)	36 (10.59%)	14 (4.12%)	134 (39.41%)		
60~	69 (20.29%)	8 (2.35%)	16 (4.71%)	7 (2.06%)	100 (29.41%)		
70~	31 (9.12%)	1 (0.29%)	14 (4.12%)	8 (2.35%)	54 (15.88%)		
**Household registration**							
Town	132 (38.82%)	16 (4.71%)	57 (16.76%)	25 (7.35%)	230 (67.65%)	3.638	0.059
Rural	75 (22.06%)	2 (0.59%)	21 (6.18%)	12 (3.53%)	110 (32.35%)		
**Religion**							
with	10 (2.94%)	3 (0.88%)	9 (2.65%)	4 (1.18%)	26 (7.65%)	4.675	0.031
Without	197 (57.94%)	15 (4.41%)	69 (20.29%)	33 (9.71%)	314 (92.35%)		
**Educational level**							
Junior Secondary and below	54 (15.88%)	5 (1.47%)	20 (5.88%)	13 (3.82%)	92 (27.05%)	2.106	0.349
Senior Secondary School and Technical Secondary School	59 (17.35%)	6 (1.76%)	26 (7.65%)	13 (3.82%)	104 (30.59%)		
Junior College and above	94 (27.65%)	7 (2.06%)	32 (9.41%)	11 (3.24%)	144 (42.36%)		
**Marital status**							
Unmarried	4 (1.18%)	0 (0.00%)	0 (0.00%)	0 (0.00%)	4 (1.18%)	/	0.008
Married	199 (58.53%)	16 (4.71%)	72 (21.18%)	34 (10.00%)	321 (94.41%)		
Divorce	3 (0.88%)	1 (0.29%)	3 (0.88%)	1 (0.29%)	8 (2.35%)		
Widowed	1 (0.29%)	3 (0.88%)	2 (0.59%)	1 (0.29%)	7 (2.06%)		
***Per capita* annual household income**							
<30,000 RMB	82 (24.12%)	12 (3.53%)	36 (10.59%)	28 (8.24%)	158 (46.47%)	14.848	0.001
30,000 RMB~	96 (28.24%)	7 (2.06%)	35 (10.29%)	10 (2.94%)	148 (43.53%)		
100,000 RMB~	29 (8.53%)	1 (0.29%)	3 (0.88%)	1 (0.29%)	34 (10.00%)		
**Tumor location**							
Upper lobe of left lung	61 (17.94%)	3 (0.88%)	17 (5.00%)	10 (2.94%)	91 (26.76%)	/	0.182
Lower lobe of left lung	32 (9.41%)	4 (1.18%)	12 (3.53%)	8 (2.35%)	56 (16.47%)		
Upper lobe of right lung	74 (21.76%)	3 (0.88%)	27 (7.94%)	9 (2.65%)	113 (33.24%)		
Middle Lobe of right lung	10 (2.94%)	0 (0.00%)	3 (0.88%)	4 (1.18%)	17 (5.00%)		
Lower lobe of right lung	24 (7.06%)	5 (1.47%)	16 (4.71%)	6 (1.76%)	51 (15.00%)		
Other	6 (1.76%)	3 (0.88%)	3 (0.88%)	0 (0.00%)	12 (3.53%)		
**Pathological type**							
Squamous cell carcinoma	21 (6.18%)	0 (0.00%)	4 (1.18%)	4 (1.18%)	29 (8.53%)	/	<0.001
Small cell carcinoma	42 (12.35%)	0 (0.00%)	6 (1.76%)	4 (1.18%)	52 (15.29%)		
Adenocarcinoma	141 (41.47%)	17 (5.00%)	62 (18.24%)	28 (8.24%)	248 (72.94%)		
Other	3 (0.88%)	1 (0.29%)	6 (1.76%)	1 (0.29%)	11 (3.24%)		
**Degree of differentiation**							
Highly differentiated	23 (6.76%)	3 (0.88%)	5 (1.47%)	3 (0.88%)	34 (10.00%)	1.442	0.965
Intermediate differentiation	30 (8.82%)	3 (0.88%)	11 (3.24%)	7 (2.06%)	51 (15.00%)		
High-intermediate differentiation	8 (2.35%)	1 (0.29%)	2 (0.59%)	2 (0.59%)	13 (3.82%)		
Low to medium differentiation	13 (3.82%)	0 (0.00%)	4 (1.18%)	2 (0.59%)	19 (5.59%)		
Low differentiation	42 (12.35%)	2 (0.59%)	17 (5.00%)	8 (2.35%)	69 (20.29%)		
Undifferentiated	11 (3.24%)	2 (0.59%)	5 (1.47%)	0 (0.00%)	18 (5.30%)		
Unknown	80 (23.53%)	7 (2.06%)	34 (10.00%)	15 (4.41%)	136 (40.00%)		
**Duration of illness**							
<6 months	42 (12.35%)	2 (0.59%)	14 (4.12%)	12 (3.53%)	70 (20.59%)	1.443	0.838
6 months~	29 (8.53%)	4 (1.18%)	11 (3.24%)	8 (2.35%)	52 (15.29%)		
1 year~	40 (11.76%)	6 (1.76%)	12 (3.53%)	2 (0.59%)	60 (17.65%)		
2 years~	49 (14.41%)	2 (0.59%)	20 (5.88%)	10 (2.94%)	81 (23.82%)		
3 years~	47 (13.82%)	4 (1.18%)	21 (6.18%)	5 (1.47%)	77 (22.65%)		
**Clinical stage**							
IA	118 (34.71%)	17 (5.00%)	52 (15.29%)	25 (7.35%)	212 (62.35%)	7.348	0.119
IB	15 (4.41%)	0 (0.00%)	8 (2.35%)	0 (0.00%)	23 (6.76%)		
IIA	12 (3.53%)	0 (0.00%)	2 (0.59%)	1 (0.29%)	15 (4.41%)		
IIB	23 (6.77%)	0 (0.00%)	8 (2.35%)	2 (0.59%)	33 (9.72%)		
IIIA	39 (11.47%)	1 (0.29%)	8 (2.35%)	9 (2.65%)	57 (16.76%)		
**VAS score**							
0 point	129 (37.94%)	29 (8.53%)	19 (5.59%)	11 (3.24%)	188 (55.29%)	29.684	<0.001
1 ~ 3 points	64 (18.82%)	6 (1.76%)	18 (5.29%)	13 (3.82%)	101 (29.71%)		
4 ~ 6 points	12 (3.53%)	7 (2.06%)	9 (2.65%)	11 (3.24%)	39 (11.47%)		
7 ~ 10 points	2 (0.59%)	2 (0.59%)	3 (0.88%)	5 (1.47%)	12 (3.53%)		
**KPS score**							
100 points	40 (11.76%)	6 (1.77%)	20 (5.88%)	5 (1.47%)	71 (20.88%)	0.872	0.640
90 points	132 (38.83%)	12 (3.53%)	47 (13.82%)	20 (5.88%)	211 (62.06%)		
Under 80 points	35 (10.29%)	0 (0.00%)	11 (3.24%)	12 (3.53%)	58 (17.06%)		
**Surgery**							
Yes	170 (50.00%)	29 (8.53%)	45 (13.24%)	20 (5.88%)	264 (77.65%)	6.115	0.013
No	37 (10.88%)	8 (2.35%)	22 (6.47%)	9 (2.64%)	76 (22.35%)		
**Chemotherapy**							
Yes	84 (24.70%)	5 (1.47%)	26 (7.65%)	9 (2.65%)	124 (36.47%)	3.856	0.051
No	123 (36.18%)	13 (3.82%)	52 (15.29%)	28 (8.24%)	216 (63.53%)		
**Radiation therapy**							
Yes	20 (5.88%)	1 (0.29%)	11 (3.24%)	3 (0.88%)	35 (10.29%)	0.229	0.715
No	187 (55.00%)	17 (5.00%)	67 (19.71%)	34 (10.00%)	305 (89.71%)		
**Targeted therapy**							
Yes	11 (3.24%)	1 (0.29%)	17 (5.00%)	4 (1.18%)	33 (9.71%)	11.647	0.001
No	196 (57.65%)	17 (5.00%)	61 (17.94%)	33 (9.70%)	307 (90.29%)		
**Treatment status**							
In progress	37 (10.88%)	4 (1.18%)	24 (7.06%)	10 (2.94%)	75 (22.06%)	63.714	<0.001
Finished	137 (40.29%)	2 (0.59%)	17 (5.00%)	12 (3.53%)	168 (49.41%)		
Not yet	33 (9.71%)	12 (3.53%)	37 (10.88%)	15 (4.41%)	97 (28.53%)		
**Smoking history**							
Never	131 (38.53%)	14 (4.12%)	55 (16.18%)	22 (6.47%)	222 (65.30%)	8.606	0.014
Smoking	9 (2.65%)	1 (0.29%)	10 (2.94%)	3 (0.88%)	23 (6.76%)		
Quit	67 (19.71%)	3 (0.88%)	13 (3.82%)	12 (3.53%)	95 (27.94%)		
**Drinking history**							
Never	155 (45.59%)	10 (2.94%)	50 (14.71%)	24 (7.06%)	239 (70.30%)	6.076	0.048
Drinking	15 (4.41%)	3 (0.88%)	10 (2.94%)	5 (1.47%)	33 (9.70%)		
Quit	37 (10.88%)	5 (1.47%)	18 (5.30%)	8 (2.35%)	68 (20.00%)		
**Family history**							
No family history of cancer	137 (40.29%)	10 (2.94%)	54 (15.88%)	25 (7.35%)	226 (66.47%)	0.669	0.716
Family history of lung cancer	51 (15.00%)	5 (1.47%)	19 (5.59%)	11 (3.24%)	86 (25.30%)		
Family history of other cancers	19 (5.59%)	3 (0.88%)	5 (1.47%)	1 (0.29%)	28 (8.23%)		
**Chronic illness**							
Without	100 (29.41%)	6 (1.76%)	43 (12.65%)	17 (5.00%)	166 (48.82%)	0.056	0.825
With	107 (31.47%)	12 (3.53%)	35 (10.30%)	20 (5.88%)	174 (51.18%)		
**Mental toughness score**							
34 points~	100 (29.41%)	3 (0.88%)	10 (2.94%)	2 (0.59%)	115 (33.82%)	72.297	<0.001
28 points~	67 (19.71%)	4 (1.18%)	30 (8.82%)	5 (1.47%)	106 (31.18%)		
21 points~	24 (7.06%)	6 (1.76%)	22 (6.47%)	8 (2.35%)	60 (17.64%)		
<21 points	16 (4.71%)	5 (1.47%)	16 (4.71%)	22 (6.47%)	59 (17.36%)		

### 3.2 Emotional state of participants

Among the 340 patients included in this study, 133 were assessed for anxiety, and the incidence rate of anxiety was 39.12%. This group included 18 patients with mild anxiety, 78 with moderate anxiety, and 37 with severe anxiety. The remaining 207 patients (60.88%) did not have anxiety disorders (Table 1).

### 3.3 Univariate analysis of influencing factors associated with anxiety in patients with early and middle-stage lung cancer

Univariate analysis showed that religion, marital status, surgical treatment, and tobacco and alcohol intake were correlated with the onset of anxiety (*p* < 0.05). Additionally, significant differences were found between the *per capita* annual family income, pathological type of cancer, VAS score, targeted therapy, treatment stage, psychological toughness, and anxiety disorders (*p* < 0.01), as shown in Table 1.

### 3.4 Multivariate analysis of influencing factors associated with anxiety in patients with early and middle-stage lung cancer

**Figure 1:**
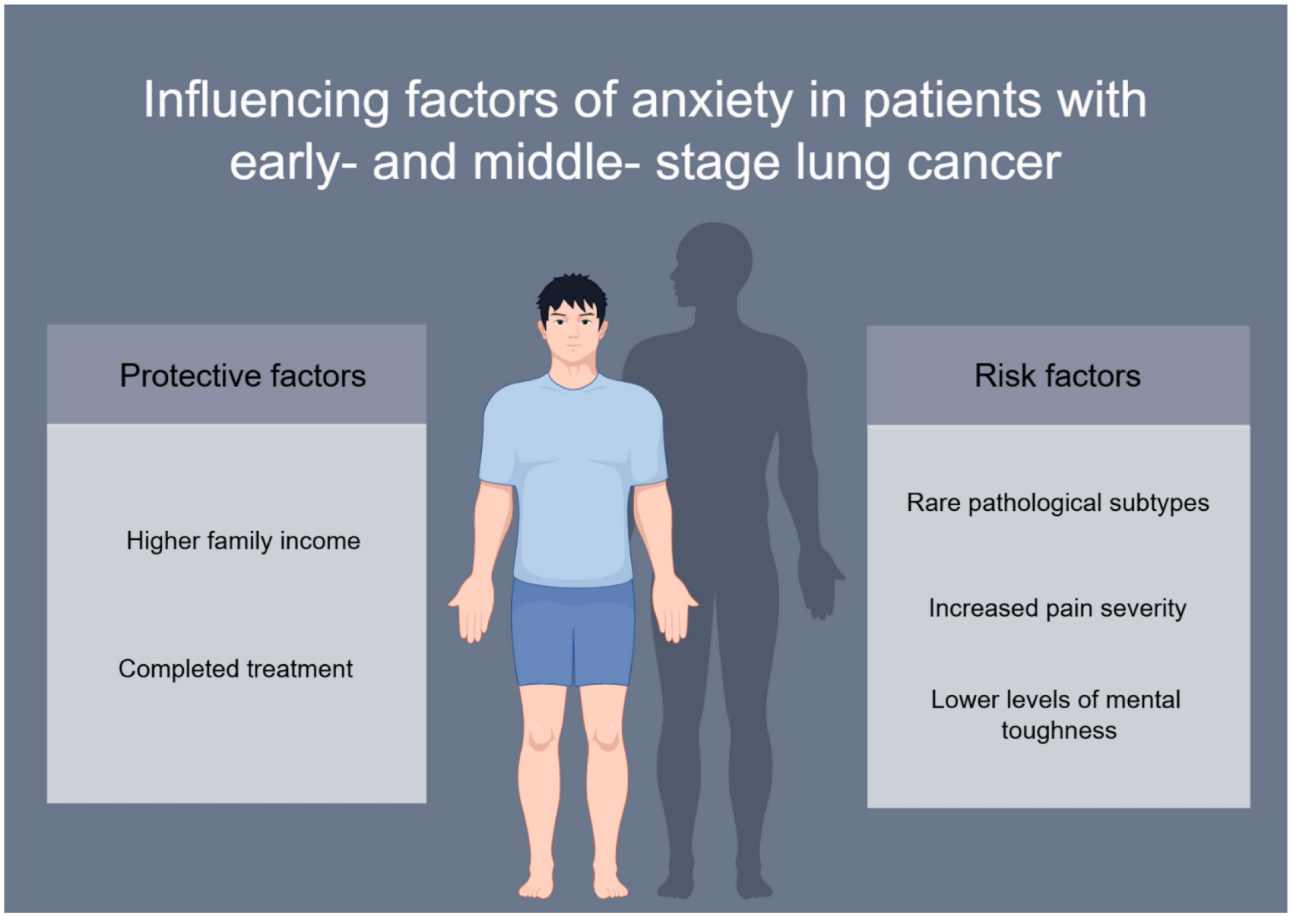
Influencing factors of anxiety in patients with early- and middle- stage lung cancer (By Figdraw).

Multivariate analysis was conducted using factors related to anxiety onset in patients with early- and middle-stage lung cancer (yes = 1, no = 0). Independent variables were selected based on their significant differences in the univariate analysis, with a value of 1 indicating the presence of a factor and 0 indicating its absence. The findings revealed that higher *per capita* family income and completed treatment independently acted as protective factors against anxiety onset in patients with early- and middle-stage lung cancer. Conversely, rare pathological types, increased pain severity, and lower levels of mental toughness were identified as independent risk factors for anxiety onset in these patients (Table 2). We visually showed the influencing factors of anxiety state in patients with early and middle stage lung cancer in Figure 1.

**Table 2:** Multivariate analysis of influencing factors of anxiety in patients with early and middle stage lung cancer.

Item	Odds ratio	95% CI	*p* value
Religion (with/without)	0.798	(0.217, 2.937)	0.734
**Marital status**			
Unmarried	0.000	(0.000, /)	0.999
Married	0.203	(0.018, 2.274)	0.196
Divorce	0.840	(0.042, 16.908)	0.909
***Per capita* annual household income**			
30,000 RMB~	0.334	(0.164, 0.681)	0.003
100,000 RMB~	0.066	(0.016, 0.283)	<0.001
**Pathological type**			
Small cell carcinoma	1.049	(0.212, 5.204)	0.953
Adenocarcinoma	3.137	(0.662, 14.863)	0.150
Other	14.924	(1.439, 154.818)	0.024
Surgery ()	0.588	(0.245, 1.410)	0.234
Targeted therapy (Yes/No)	2.286	(0.710, 7.357)	0.166
**Treatment status**			
Finished	0.177	(0.065, 0.486)	0.001
Not yet	2.136	(0.714, 6.397)	0.175
**VAS score**			
1 ~ 3 points	1.311	(0.637, 2.698)	0.462
4 ~ 6 points	5.886	(1.910, 18.137)	0.002
7 ~ 10 points	18.331	(2.709, 124.050)	0.003
**Smoking history**			
Smoking	1.916	(0.339, 10.826)	0.462
Quit	1.185	(0.450, 3.120)	0.731
**Drinking history**			
Drinking	1.134	(0.301, 4.270)	0.852
Quit	1.495	(0.603, 3.707)	0.386
**Mental toughness score**			
28 points~	2.647	(1.149, 6.096)	0.022
21 points~	10.049	(3.727, 27.091)	<0.001
<21 points	12.278	(4.755, 31.703)	<0.001

## 4 Discussion

### 4.1 High prevalence of anxiety among patients with early and middle-stage lung cancer

This study investigated the prevalence of anxiety and related risk factors in patients with early- and middle-stage lung cancer using a questionnaire. The prevalence of anxiety among 340 participants was 39.12%. The results of the multivariate logistic regression analysis showed that higher family income and completed treatment were independent protective factors for the onset of anxiety, while rare pathologic types, higher degree of pain, and lower psychological toughness were independent risk factors for the onset of anxiety in patients with early- and middle-stage lung cancer. A previous study showed that anxiety was associated with an increased risk of lung cancer, cancer-specific death, and all-cause death (Vodermaier et al., 2017; Wang et al., 2020). Therefore, it is necessary to effectively identify groups at a high risk of anxiety.

The prevalence of anxiety reported in our study was consistent with findings from previous literature. Peh et al. (2017) found that the prevalence of anxiety was 39.6% among 144 newly diagnosed patients with cancer who had many similar characteristics to those with early- and middle-stage lung cancer included in our study. Yang et al. (2021) reported that the incidence of anxiety among patients with cancer during the COVID-19 pandemic was 34.9%. Another study that included patients with different types of cancers or hematologic malignancies showed that 35.2% of participants showed symptoms of anxiety (Zeilinger et al., 2022). A study focusing on hospitalized patients with cancer reported a lower incidence of anxiety (22%) (van den Brekel et al., 2020). A possible reason for this is that the hospitalized patients received better medical care and distressing symptoms were alleviated to some extent, which reduced the incidence of adverse emotions. Interestingly, a cross-sectional study of patients with advanced cancer receiving palliative care reported that the prevalence of anxiety was only 25.7% (Gontijo Garcia et al., 2023). Advanced patients often learn to cope with cancer after multiple anti-cancer treatments, which may partly explain the lower rates of anxiety. In addition, these studies were conducted in hospitals practicing Western medicine, whereas patients in our study, who presented to hospitals practicing Chinese medicine, often received multiple treatments with poor outcomes, which may explain the difference in results.

### 4.2 Protective factors influencing anxiety onset in patients with early and middle-stage lung cancer

Previous studies have reported that patients with lung cancer have the highest incidence of anxiety among those with cancer, indicating that mental health issues in patients with lung cancer should be taken seriously (Linden et al., 2012). Our results suggest that higher household income and treatment completion are independent protective factors against anxiety in patients with early- and middle-stage lung cancer. A study on anxiety disorders in long-term survivors 5 and 10 years after cancer diagnosis concluded that financial hardship was strongly associated with the onset of anxiety (Götze et al., 2020). Patients undergo surgery, radiotherapy, chemotherapy, and other multi-course treatments after being diagnosed with malignant tumors. Low-income individuals find it more difficult to seek medical treatment, and high expenses tend to increase their psychological burden, resulting in different degrees of anxiety. Notably, previous literature has shown that patients with cancer who experience anxiety have a higher risk of emergency department visits and hospitalizations, longer hospital stays, and higher medical costs (Mausbach et al., 2020). This may contribute to a vicious cycle that is detrimental to patients and their families.

Our study found that although surgery and targeted therapy influenced the onset of anxiety in the univariate analysis, they were no longer influencing factors after the confounding factors were removed in the multivariate analysis. Most current studies have only analyzed the effects of treatment without distinguishing between ongoing treatment, completed treatment, and no treatment. Patients undergoing treatment are often plagued by drug toxicity and side effects, such as lung injury caused by radiotherapy, vomiting, myelosuppression caused by chemotherapy, diarrhea, and skin lesions caused by targeted therapy, leading to negative emotions, such as anxiety. When treatment programs are stopped for reasons, such as organ damage, serious drug toxicity, or side effects, patients may experience anxiety and helplessness. Researchers have reported that anxiety levels among patients with cancer increased during the COVID-19 pandemic due to disrupted and delayed treatment (Yildirim et al., 2021).

### 4.3 Risk factors influencing anxiety onset in patients with early and middle-stage lung cancer

Our findings indicate that rare pathological subtypes, higher levels of pain, and lower levels of mental resilience are independent risk factors for anxiety in patients with early- and mid-stage lung cancer. The clinical symptoms, treatment methods, and survival prognoses of patients with different pathological types of lung cancer vary significantly. Rare tumor types tend to exhibit higher malignancy, increased infiltration and metastatic capabilities, and reduced sensitivity to radiotherapy and chemotherapy. Consequently, these factors contribute to heightened anxiety in affected individuals. Clinicians and psychologists should pay more attention to patients with rare types of tumors. However, there is a gap in the research on the relationship between the pathological types of lung cancer and the onset of anxiety in patients. Therefore, high-quality multicenter studies are required.

Pain is a common symptom in patients with cancer. A multicenter cross-sectional survey using patient-reported outcomes found that pain ranked fourth (21.1%) among all symptoms in patients with cancer in China (Tang et al., 2020). Most previous studies have reported pain as a risk factor for anxiety onset. A study conducted in patients with tumors undergoing chemotherapy determined that severe pain was associated with higher levels of depression, anxiety, and cognitive dysfunction (Shin et al., 2022). Another study of 156 patients with cancer who had chronic pain found that pain acceptance was inversely associated with depression and anxiety (Xu et al., 2019). Studies on patients with breast cancer (Shabangu et al., 2023), multiple myeloma (Shi et al., 2022), and endometrial cancer (Wang et al., 2020) reported similar results. Therefore, healthcare providers should actively and effectively relieve pain to improve mental health and quality of life (Maindet et al., 2019).

The CD-RISC-10 is an appropriate tool for measuring resilience, with the advantages of being rapid and convenient. It has been validated in patients with breast cancer and parents of children with cancer (Alarcón et al., 2020; Ye et al., 2017). The mainstream view is that mental health is positively correlated with resilience (Hu et al., 2023; Matzka et al., 2016), and our findings are consistent with this. These results suggest that we can improve the mental toughness of patients through various types of resilience training to relieve anxiety and enrich treatment methods. Studies have shown that training methods, such as cognitive therapy and music therapy, can relieve anxiety and depression in cancer survivors (Eseadi and Ngwu, 2023; Nissen et al., 2020). A systematic review and meta-analysis found that mindfulness-based interventions may be effective in reducing anxiety, depression, and fatigue in lung cancer patients (Li et al., 2023). However, standardized procedures and evaluation criteria for these training methods need to be established. Research on the level of mental toughness among patients with cancer is still in its early stages. Our study fills the gap in this field among patients with early- and middle-stage lung cancer.

### 4.4 Strengths and limitations

We conducted a cross-sectional examination of the psychological status of patients with early- and middle-stage lung cancer. The findings of this study may help improve both the quality of life and long-term prognosis. Furthermore, our results suggest that healthcare professionals, psychologists, and family members should pay attention to patients’ mental health, address negative emotions, and diversify treatment approaches. The limitations of this study include the following. (1) Due to constraints associated with survey data availability, only certain factors regarding anxiety onset were analyzed. Future studies should collect more comprehensive clinical data for a more thorough analysis. (2) Given the limitations of resources, subjective self-rating scales were employed as the sole means of assessing anxiety levels among the participants. In future research, professional psychiatric diagnostic interviews should be incorporated alongside objective indicators, such as laboratory tests, to provide a more accurate reflection of anxiety incidence among patients with early- and middle-stage lung cancer.

### 4.5 Summary

In conclusion, the incidence of anxiety among Chinese patients with early- and middle-stage lung cancer was 39.12%. Higher family income and completed treatment were independent protective factors against the onset of anxiety. Rare pathological types, higher degree of pain, and lower mental toughness were independent risk factors for anxiety. Economic income determines, to some extent, the medical resources that patients can access. High treatment costs increase the risk of disease-related poverty. Therefore, the government should improve the healthcare system to alleviate the financial burden on patients. For patients receiving treatment, clinicians should promptly deal with side effects and adverse reactions. For patients in poor conditions who cannot tolerate treatment, efforts should be made to alleviate their symptoms and improve their quality of life. It is necessary to popularize the knowledge of diseases among patients with rare tumors to help them understand their diseases correctly. In addition, more care and encouragement should be provided to help build confidence in fighting the disease. Most cancer patients experience chronic pain. Scientific and standardized control of pain symptoms according to the principle of the three-ladder analgesia can effectively improve the quality of life of patients and relieve mental disorders. Good mental resilience protects against anxiety. A variety of resilience training programs are helpful in relieving anxiety and can enrich treatment methods for patients with cancer.

## Data Availability

The original contributions presented in the study are included in the article/Supplementary material, further inquiries can be directed to the corresponding author.
